# Taxonomic and functional nestedness of bird communities in urban parks of Liuzhou, China

**DOI:** 10.3897/BDJ.13.e154385

**Published:** 2025-05-21

**Authors:** Binqiang Li, Nehafta Bibi, Shanjun Ma, Wenxuan Chen, Miaodan Yang, Na Xiang, Qingjiang Cui, Lifeng Tan

**Affiliations:** 1 College of Forestry, Southwest Forestry University, Kunming, China College of Forestry, Southwest Forestry University Kunming China; 2 Planing and Design Institute, Yunnan Forestry Technological College, Kunming, China Planing and Design Institute, Yunnan Forestry Technological College Kunming China; 3 Department of Zoology, Government Girls Degree College #1 Mansehra, Mansehra, Pakistan Department of Zoology, Government Girls Degree College #1 Mansehra Mansehra Pakistan; 4 College of Forestry, Guangxi Eco-engineering Vocational & Technical College, Liuzhou, China College of Forestry, Guangxi Eco-engineering Vocational & Technical College Liuzhou China; 5 College of Resources Environment and Chemistry, Chuxiong Normal University, Chuxiong, China College of Resources Environment and Chemistry, Chuxiong Normal University Chuxiong China

**Keywords:** urbanisation, taxonomic diversity, species richness and composition, nestedness, functional diversity, Liuzhou

## Abstract

Urbanisation significantly impacts the composition and distribution of species through habitat loss and fragmentation. Nestedness is a significant pattern often observed in species assemblages on islands or within fragmented systems. However, numerous studies on nestedness have focused on species richness and composition, neglecting the role of species traits in generating and explaining nestedness. To determine whether functional nestedness follows the same pattern as taxonomic nestedness. In this study, we examined the nestedness patterns of bird assemblages (all birds, passerines, insectivorous, omnivorous and resident birds) across 17 urban parks in Liuzhou of Guangxi Province, China, focusing on taxonomic and functional nestedness. From April 2021 to February 2022, we conducted line transect surveys of bird communities, with three surveys during the breeding season and three surveys during the non-breeding season. In total, we documented 95 bird species. Taxonomic nestedness was assessed using NODF (a nestedness metric, based on overlap and decreasing fill) and WNODF (weighted nestedness metric, based on overlap and decreasing fill) metrics, while functional nestedness was evaluated using treeNODF. Our results showed that none of the birds, passerines, insectivorous, omnivorous and resident birds in Liuzhou urban parks exhibited significant nestedness patterns. However, the nested pattern strongly depended on the choice of the null model. In contrast, as the park area gradually decreases, we observed significant functional nestedness, implying that the trait loss in parks with decreasing area follows an ordered pattern, where smaller parks represent nested subsets of functional traits found in larger parks. From the perspective of species conservation, we recommend prioritizing the protection of larger urban parks to support species with larger habitat requirements. All in all, our findings highlight the importance of considering both taxonomic and functional nestedness in urban biodiversity conservation.

## Introduction

Urbanisation is one of the primary drivers of land-use change (McKinney 2002). Urban areas consist of extensive impervious surfaces, alongside various greenbelts, water bodies, wetlands and other natural spaces. Green spaces have distinct environmental characteristics that set them apart from built-up areas (McKinney 2002, Tan et al. 2020). Due to their relative isolation and the distinct boundaries that separate them from the surrounding urban environment, the biotic communities of urban green spaces are expected to exhibit similarities to those of oceanic islands (Fernández‐Juricic and Jokimäki 2001, Fattorini et al. 2017). This is particularly evident when considering factors such as the limited species exchange, the development of specific ecological niches and the unique adaptations which organisms in these isolated habitats must undergo to survive and thrive (Fattorini et al. 2017). Urban land-change threatens biodiversity and alters species composition and distribution through loss of habitat (McKinney 2008, Wang et al. 2013, Nielsen et al. 2013). Urban green spaces can serve as valuable biodiversity reserves and there is growing interest in incorporating them into urban planning and biodiversity conservation efforts (Fattorini et al. 2017, Sol et al. 2020). Urban parks have a large amount of green space. However, numerous studies have focused on community composition (e.g., species richness) in urban parks, while the specific patterns of community composition, such as nestedness, remain relatively poorly understood.

Nestedness is a common pattern often observed in faunal assemblages on islands and fragmented habitats. This concept was initially proposed by Darlington (1957) and later formally defined and popularised by Patterson and Atmar (1986). Nestedness describes a situation where the species found in species-poor sites form a true subset (Wright et al. 1997, Fischer and Lindenmayer 2005, Almeida‐Neto et al. 2008). In other words, the species assemblages exhibit an orderly, nested distribution pattern across different habitats. Perfect nestedness occurs when all species-poor sites are subsets of the assemblages found in species-rich sites (Patterson and Atmar 1986, Almeida‐Neto et al. 2008). This concept is crucial in ecological studies as it provides insight into species distributions across varying habitats. Understanding nestedness allows researchers to predict how these distributions may shift in response to environmental changes and ecological disturbances (Kadmon 1995, Patterson 2005, Matthews et al. 2015a, Tan et al. 2020).

Numerous empirical studies have demonstrated that variations in habitat characteristics, such as isolation, size, quality and the presence of nested habitats, along with species attributes like area requirements, abundance and tolerance to abiotic factors, are the primary drivers of nestedness in metacommunities (Almeida‐Neto et al. 2008, Matthews et al. 2015a). Nested patterns are not necessarily significant because significance depends on the choice of the null model (Almeida-Neto and Ulrich 2011). In fact, using inconsistent methods and null model metrics can lead to overestimation of the importance of nesting in an ecosystem or can lead to inaccurate predictions (Cook and Quinn 1998, Almeida-Neto and Ulrich 2011). The empirical data analysis indicates that two metrics of nestedness, matrix temperature and the discrepancy measure, generally overestimate nestedness levels in metacommunities (Almeida‐Neto et al. 2008). Matthews et al. (2015a) analysed 97 datasets from published studies on habitat islands, using the NODF metric (a nestedness metric, based on overlap and decreasing fill) to assess nestedness. Their findings revealed that significant nestedness is much less common (only 9%) in habitat islands than previously reported (Matthews et al. 2015a). Moreover, to our knowledge, there are at least four null models used for nested pattern analysis in previous studies (Zhou et al. 2012, Wang et al. 2013, Tan et al. 2020). Such as pp null mode (proportional row and proportional column constraints), rc null model (resampling with row/column weights fixed), aa null model (resampling with total weight fixed), and ss null model (resampling with total species richness fixed) (Almeida-Neto and Ulrich 2011). Different null models exhibit varying levels of constraint on matrix properties, which can lead to differences in the detection of nested patterns.

In urban bird studies, previous studies have demonstrated distinct nested patterns, while a smaller number of studies have observed anti-nestedness patterns or identified no significant nestedness at all (Fernández-Juricic 2004, Platt and Lill 2006, Zhou et al. 2012, Wang et al. 2013, Tan et al. 2020, Tian et al. 2022, Wang et al. 2023, Wang et al. 2024). Consequently, the findings of previous studies were inconsistent and continue to be debated. Moreover, limited research has been conducted on the nestedness of various bird groups, such as insectivorous, omnivorous, passerines, and resident birds. Generally, forest passerines, insectivorous birds, and resident birds are more vulnerable to habitat fragmentation (Santos et al. 2002, Fernández-Juricic 2004, Marcacci et al. 2021, Pharr et al. 2023). For instance, as the degree of urbanisation increases, the richness of insectivorous birds declines (Pal et al. 2019). Insectivorous birds tend to inhabit intact and highly connected patches, while omnivorous birds have better adaptability to the fragmentation caused by urbanisation (Wu et al. 2024). Importantly, previous studies on nestedness and metacommunity structure have considered species as equivalent and independent units in ecological and evolutionary terms (Melo et al. 2014, Chen et al. 2022, Millien et al. 2024). Consequently, traditional nested metrics overlook key ecological relationships (Melo et al. 2014). For instance, species that share most functional traits may be mostly redundant for a particular ecological process or reveal a higher niche overlap (Melo et al. 2014). To address this limitation, Melo et al. (2014) introduced treeNODF, a method that extends the NODF index to systems where the similarity of descriptor variables is represented by a tree-like structure. The computation of treeNODF is similar to that of NODF, but it utilises branch lengths instead of the sum of species occurrences (Melo et al. 2014).

In this study, we examined the nested distribution of bird assemblages in 17 urban parks in Liuzhou, China. Our study has three main objectives as follows: (1) to assess whether bird assemblages in Liuzhou urban parks conform to the nested subset pattern; (2) to evaluate how different null models influence the robustness of nested patterns; (3) to determine whether functional nestedness follows the same pattern as taxonomic nestedness. After revealing the key processes of nestedness, we can inform conservation strategies and urban planning efforts aimed at preserving bird biodiversity in urban areas.

## Material and methods

### Study area

**Figure 1.**
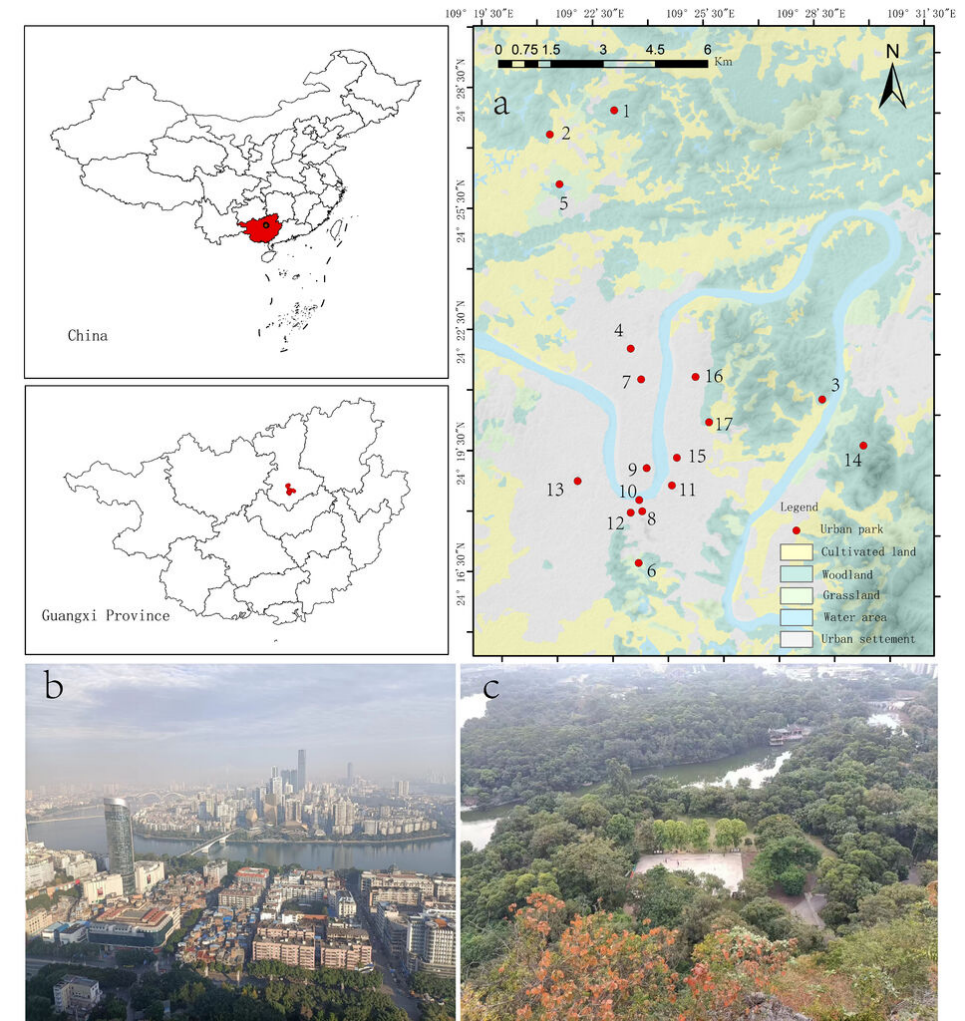
The location of 17 urban parks in Liuzhou of Guangxi, China; **a** the location of urban parks; **b** urban landscape; **c** park view.

Our study was conducted in Liuzhou (108°50'-109°44'E, 23°54'-24°50'N), which is a well-known industrial city located in Guangxi Province, China (Fig. 1). As of 2021, the population of Liuzhou was approximately 4.1753 million. Compared to major cities, Liuzhou is considered a small to medium-sized city. It has a total land area of 1,859,600 ha, which includes 346,600 ha of cultivated land, 48,100 ha of garden land, 1,105,500 ha of forest land, 114,800 ha of grassland and 70,300 ha of industrial land (http://www.lzdqw.gov.cn). The altitude is between 100 and 200 m. The climate is subtropical monsoon, with an average annual temperature of 21.3℃ and annual precipitation of 1047.3 mm. It is hot and humid in summer and cold in winter (Li et al. 2023). Liuzhou, the largest industrial city in Guangxi, has the greatest industrial ratio in Guangxi, accounting for one-quarter of the total industry in Guangxi (http://www.lzdqw.gov.cn). Due to the rapid and unregulated industrial development, the natural vegetation in urban areas has suffered significant and irreversible damage. This has not only led to the direct loss of biodiversity but also disrupted the ecological balance and the intricate web of life that these natural habitats supported.

### Bird surveys

Firstly, all the parks have carried out boundary demarcation and sample selection. The parks we studied are all public green spaces that are planned and managed by the Liuzhou Government. Except for Junwu Forest Park (location 1 on the map), Sanmenjiang Forest Park (location 3 on the map), and Gutingshan Forest Park (location 14 on the map) (Fig. 1), which do not have park fences, the other parks are enclosed by fences. Junwu Forest Park, Sanmenjiang Forest Park, and Gutingshan Forest Park are separated from different urban areas (such as roads and buildings) by distinct boundaries, which enables them to be treated as relatively independent areas for bird sampling. When selecting parks, we also considered their functions and ecological characteristics. We mainly focused on forest-type parks. Although some parks have waterbirds, we excluded the waterbird data from our analysis.

We used the line-transect method to survey bird communities in 17 urban parks (Wilson et al. 2000) (Fig. 1). Our sampling effort in each park was approximately proportional to the logarithm of the park's area and line transects were designed to cover as many habitats as possible. We established one to two transect lines in each park, measuring 1 - 2 km in large parks and 0.2 - 1 km in smaller ones. In total, there were 21 transect lines in our study. The transect lines were positioned along park trails, each crossing the centre of the park. Our bird surveys were conducted from April 2021 to February 2022, with six surveys per park — three during the breeding season and three during the non-breeding season. For each transect, our bird surveys were conducted from 6:00 AM to 10:00 AM and from 5:00 PM to 7:00 PM again. These time slots were selected to maximise our chances of observing avian activity. During our surveys, we utilised binoculars to identify and record the species of birds observed or heard within a 100 m width on both sides of the transect, along with their individuals. We only included bird observations that were within the boundaries of the parks. Observations made outside the park boundaries, even within the 100 m width, were excluded from the analysis. The surveys were only conducted in favourable weather conditions, as we avoided them during adverse weather, such as fog or rain. Our bird identification methodology follows the established work of Zheng (2023).

### Data analysis

To evaluate the nested patterns of different bird groups, we classified birds into all birds, passerines, resident birds, insectivorous birds, and omnivorous birds. Due to the sample size limitations of non-Passeriformes, carnivores, nectarivorous, granivores, and migratory birds, these groups were not included in the nested pattern analysis. The taxa of bird species and their migratory status are based on Zheng (2023). The feeding guilds are referenced from the work of Wang et al. (2021). To evaluate species inventory completeness, we based the method on rarefaction and extrapolation of bird diversity to assess sample coverage using R package iNEXT (Chao et al. 2014). Sample coverage values range from 0 to 1, with a value greater than 0.90 indicating that the sampling is generally adequate (Chao et al. 2014, Hsieh et al. 2016). Detecting rare or cryptic species is challenging, leading to nestedness patterns that may result from passive sampling (Cutler 1994, Higgins et al. 2006). Therefore, we utilised a random placement model to assess whether the nestedness of bird assemblages in urban parks resulted from passive sampling (Coleman 1981, Tan et al. 2020). If more than 1/3 of the observed values are not within one standard deviation (SD) of the prediction curve, the passive sampling hypothesis will be rejected (Coleman 1981, Tan et al. 2020). The random placement mode was implemented in R using the code developed by Matthews et al. (2015a). We used ArcGIS 10.7 (ESRI) to measure park areas. The areas of the park were selected as factors influencing the nestedness pattern (Tan et al. 2020, Tian et al. 2022). Large parks usually have more habitat types and resources, thus allowing high levels of species richness, phylogenetic and functional diversity (Melo et al. 2014, Matthews et al. 2015a, Tan et al. 2020).

We used NODF and WNODF (weighted nestedness metric, based on overlap and decreasing fill) to measure the nestedness of bird assemblages (Almeida‐Neto et al. 2008, Almeida-Neto and Ulrich 2011). WNODF enhances the NODF metric. One of its main advantages over NODF is its ability to incorporate species abundance rather than solely relying on species richness to quantitatively assess the degree of nestedness (Almeida-Neto and Ulrich 2011, Tan et al. 2020). In addition, WNODF is able to estimate the nestedness of a matrix by analysing species (WNODFr, species in rows) and sites (WNODFc, sites in columns) separately (Almeida-Neto and Ulrich 2011, Wang et al. 2013). We utilised the pp null mode, rc null model, aa null model and ss null model provided by WNODF to quantify the nested patterns of bird communities (all birds, passerines, insectivorous, omnivorous and resident birds). It randomly generated 1,000 matrices and estimated the statistical results within the 95% confidence interval (Tan et al. 2020). Our calculations were conducted using the NODF version 2.0 programme.

We utilised treeNODF to assess the functional nestedness of birds. Like traditional NODF, treeNODF can be divided into two components (treeNODF = S.Fraction + topoNODF) (Melo et al. 2014, Chen et al. 2022): (1) S.Fraction, which represents the species composition component of treeNODF (Melo et al. 2014, Chen et al. 2022); and (2) topoNODF, which reflects the contribution of the tree topology or functional differences between species (Melo et al. 2014, Chen et al. 2022). These two components can help determine whether the observed functional nestedness is primarily driven by compositional nestedness or by tree topology (Chen et al. 2022). We used data on body size and wing length from Wang et al. (2021) for the functional trait. These characteristics effectively illustrate how bird species utilise and compete for resources, as well as their dispersal and colonisation abilities (Luck et al. 2012).

We calculated the distance between species traits using the Gower distance and converted it into a functional dendrogram with the UPGMA clustering algorithm (Chen et al. 2022). Similarly, we conducted an analysis to evaluate the similarities in the park area (Gower distance and UPGMA). Based on Melo et al. (2014), we calculated traitNODF and its components by ordering the rows in the community matrix according to decreasing park area (Melo et al. 2014) and used the simple permRows (randomly re-orders rows of the matrix and calculates the treeNODF values for each rows-re-ordered matrix) algorithm that randomises rows (parks in our case, 99 replications) to test whether the observed values of our metrics could be produced by chance (Melo et al. 2014).

## Results

### Bird species richness

**Figure 2.**
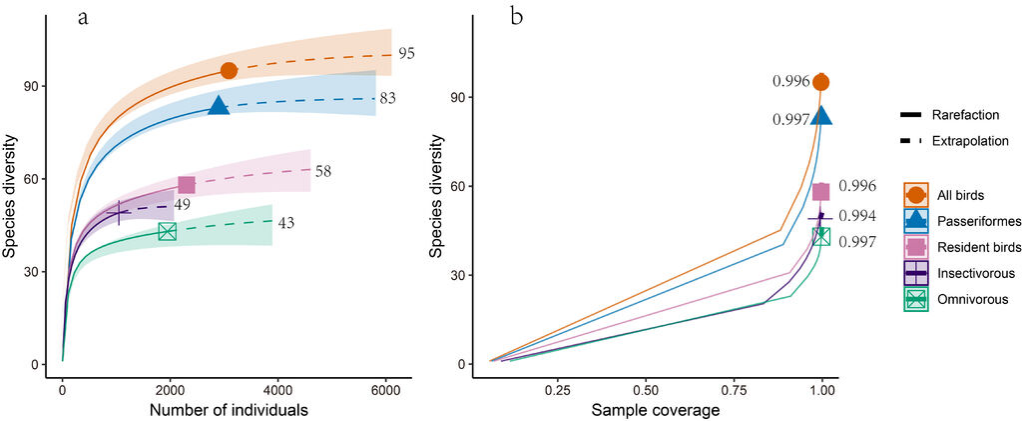
The rarefaction and extrapolation of bird species diversity in 17 urban parks in Liuzhou. **a** species richness; **b** sample coverage; the solid lines represent rarefaction, while the dashed lines represent extrapolation; shaded regions, 95% confidence intervals.

We recorded 95 bird species in Liuzhou urban parks (Suppl. material 1). The species richness observed in each park ranged from 9 to 60. Additionally, the species' (all birds, passerines, resident, insectivorous, and omnivorous) rarefaction and extrapolation curve approached an asymptote (Fig. 2a), the sample coverage of all groups exceeded 0.90 (Fig. 2b), indicating a high level of completeness in the bird inventory across 17 parks.

### Taxonomic nestedness of bird assemblages

**Figure 3.**
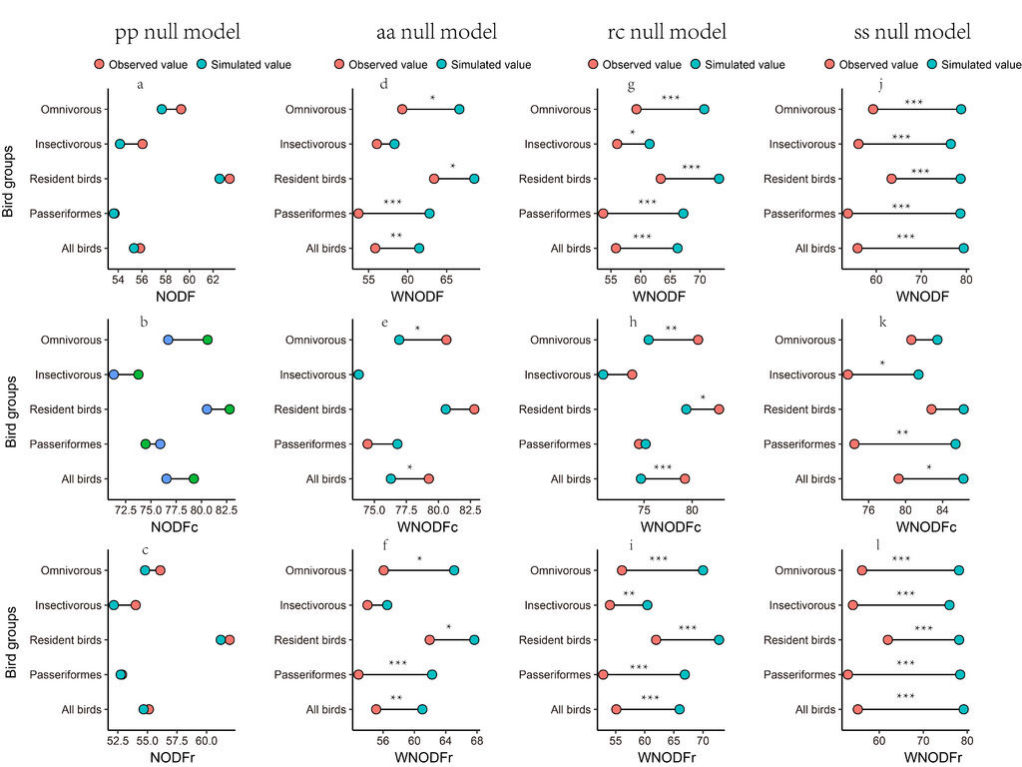
The taxonomic nestedness of bird assemblages in Liuzhou. The taxonomic nestedness (NODF/WNODF) can estimate the nestedness of a matrix by analysing species (NODFr/WNODFr, species in rows) and sites (NODFc/WNODFc, sites in columns) separately. *P < 0.05, **P < 0.01, ***P < 0.001.

In the analysis of the species-by-site matrix, the NODF analysis showed no significant nestedness patterns for all bird groups (Fig. 3a). However, the WNODF analysis highlighted some notable differences depending on the null model used. Under the aa null model, except insectivorous species, other groups displayed significant anti-nestedness patterns (Fig. 3d). However, the rc (Fig. 3g) and ss null models indicated that all bird groups showed significant anti-nestedness patterns (Fig. 3j).

Across sites, differences were observed in the results for NODFc and WNODFc (species composition). For example, the pp null models showed no significant nestedness or anti-nestedness patterns for NODFc (Fig. 3b). The aa null model results showed that, except for omnivorous and all birds, the species composition of other groups did not exhibit significant anti-nestedness patterns (Fig. 3e). The rc null model analysis revealed that, apart from passerines and insectivorous, the species composition of other groups exhibited significant anti-nestedness patterns (Fig. 3h). However, the ss null model analysis indicated that, except for resident and omnivorous, other groups showed a significant anti-nestedness pattern (Fig. 3k).

**Figure 4.**
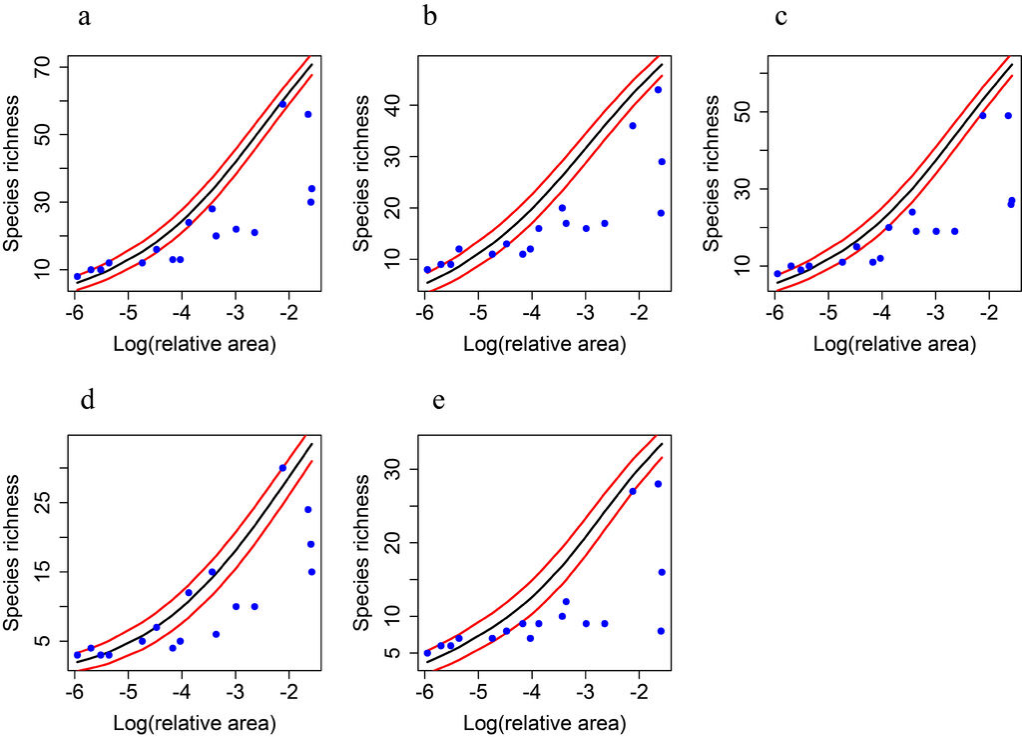
Comparison of observed data to expected values under the random placement model for birds in 17 urban parks in Liuzhou. Expected values (black line) and associated standard deviations (red lines) are shown; filled points represent observed species richness; **a** all birds; **b** Passeriformes; **c** insectivorous; **d** omnivorous; **e** resident birds.

Likewise, differences were observed in the results for NODFr and WNODFr (species incidence). The pp null model results showed that all bird groups had no significant nestedness or anti-nestedness patterns (Fig. 3c). The aa null model results indicated that, except for insectivorous, other groups showed a significant anti-nestedness pattern (Fig. 3f). Under the rc (Fig. 3i) and ss null models (Fig. 3l), all bird groups exhibited significant anti-nestedness patterns. The random placement model confirmed that the observed nestedness or anti-nestedness of all bird groups in Liuzhou urban parks was not caused by passive sampling (Fig. 4a-Fig. 4e).

### Functional nestedness

**Figure 5.**
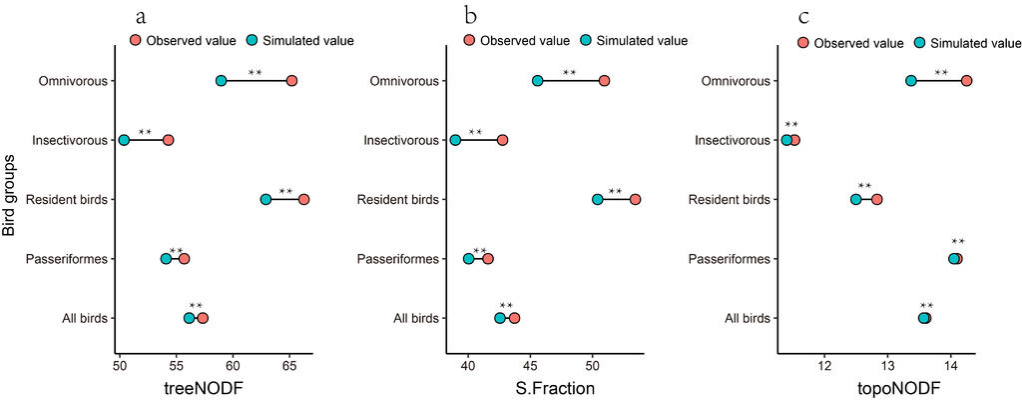
The functional nestedness of bird assemblages in Liuzhou. The functional nestedness (traitNODF) and its two components (S.Fraction and topoNODF) were calculated by ordering the species matrix with a selected gradient (park area); **a** treeNODF; **b** S.Fraction; **c** topoNODF; *P < 0.05, **P < 0.01, ***P < 0.001.

For the species-by-site matrix ordered by decreasing park area, the observed values of treeNODF (Fig. 5a) and its components (S.Fraction and topoNODF) were significantly higher than expected by chance (*z* = 4.55-5.68, *p* = 0.01) (Fig. 5b-Fig. 5c). As a result, the functional diversity in small parks was not only lower than that in large parks, but it also represents a nested subset of the diversity found in those larger areas. Notably, the observed treeNODF values were primarily driven by S.Fraction (42.76 - 53.44) rather than the topoNODF component (11.52 - 14.25).

## Discussion

### Taxonomic diversity

In the present study, we conducted a systematic analysis of the nestedness characteristics of bird assemblages in Liuzhou, China. Our findings reveal that the bird assemblage in Liuzhou urban park has no significant nestedness pattern. Our findings differ from previous studies. For example, nested patterns of bird assemblages were evident in urban parks in Madrid (Fernández-Juricic 2004), Melbourne (Platt and Lill 2006), Hong Kong (Zhou et al. 2012), Hangzhou (Wang et al. 2013), Nanjing (Tan et al. 2020) and Zhengzhou (Wang et al. 2024). However, the urban forest bird assemblage in Guiyang (Zheng et al. 2021), Sanya and Haikou (Tian et al. 2022) showed an anti-nested pattern. On the one hand, most previous studies focused on the breeding birds. Due to the limitations of our bird sampling (three times in the breeding season and three times in the non-breeding season), we did not analyze the nestedness patterns of birds in the breeding and non-breeding seasons separately. Bird assemblages can show different nestedness patterns across seasons. For instance, they exhibit nestedness during the non-breeding season but not during the breeding season (Chen et al. 2017). The difference between our findings and previous studies may result from our inability to analyze the seasonal nestedness patterns of bird assemblages separately. On the other hand, we observed considerable variation in the outcomes of different null model analyses. In general, the most controversial aspect of nestedness analysis is selecting the appropriate null model to determine whether the nestedness pattern is significant (Ulrich et al. 2017). We examined four well-known null models that have been used in previous studies. These models apply different constraints to the rows and columns of the species-sites matrix, potentially resulting in varying outcomes depending on their processing methods (Wang et al. 2023). To date, ecologists have not reached a consensus on how to measure the degree of nestedness or how to choose the null model (Wang et al. 2023). The absence of a consensus amongst researchers has significantly hindered the integration of knowledge related to the universal patterns of nestedness and the complex mechanisms that drive these patterns (Ulrich et al. 2017). To address this issue, we recommend employing at least two null models to minimise the risk of type I errors.

We observed variations in the results from four different null models when focusing exclusively on species composition. For example, the pp null model indicated that none of the bird groups exhibited a significant nested pattern. In contrast, the other three null models suggested that all bird groups displayed a significant anti-nested pattern, although substantial differences were noted among passerines, resident, insectivorous, and omnivorous. Furthermore, when examining species incidence, the results of the rc and ss null models showed that all bird groups exhibited significant anti-nested patterns. These findings highlight the complexity of nestedness patterns in bird assemblages and underscore the variability introduced by different null models and bird groups. We propose that nested patterns in urban bird communities may not be as prevalent as previously thought, and this observation could be significantly influenced by the methodologies employed. According to WNODF, for each observed and expected index, if the observed value is significantly greater than the expected value, then we can assume that the assemblage of species is nested (Almeida-Neto and Ulrich 2011). If the observed value is significantly lower than the expected value, then we can assume that the species assemblage is anti-nested (Almeida-Neto and Ulrich 2011, Matthews et al. 2015a). Previous studies conducted in Hangzhou (Wang et al. 2013), Nanjing (Tan et al. 2020) and Zhengzhou (Wang et al. 2024) revealed significantly lower observations than expected. Consequently, these studies may need to reconfirm the robustness of their results. In fact, it is uncommon for natural communities to show significant nestedness. For example, previous studies on islands show that 16% of study cases have anti-nested patterns and only 9% have nested patterns (Matthews et al. 2015a). It implies that previous conservation recommendations, based on the assumption of significant nestedness in most fragmented landscapes, may need to be re-evaluated (Matthews et al. 2015a).

We observed that the bird assemblage in Liuzhou urban park exhibits a significant anti-nestedness pattern. In the anti-nested pattern, species are always absent from sites that are richer than the most depauperate site in which they occur (Poulin and Guégan 2000, Almeida‐Neto et al. 2008). In fact, a nested pattern does not exist if all species have the same incidence and all sites have the same species richness (Almeida-Neto and Ulrich 2011). Similarly, there will be no nestedness if there is no overlap in species ranges or if no same species is found in at least two different sites (Almeida-Neto and Ulrich 2011). Based on beta diversity (variation of the species composition of assemblages), if the species composition of each site is unique and lacks common species, this may also lead to non-nested or anti-nestedness, that is, species replacement between different sites (Almeida‐Neto et al. 2008, Baselga 2009). Previous studies have indicated that the breeding birds in the urban parks of Liuzhou are primarily characterised by turnover components, with noticeable species replacements occurring amongst the parks (Li et al. 2023). Generally, urbanisation is the primary factor influencing changes in bird beta diversity. The variation in species composition shows an increasing trend as urban impervious areas expand (Marcacci et al. 2021). Therefore, from the perspective of the impact of urbanisation on species composition, the high turnover of species may lead to the anti-nestedness pattern of urban park birds. Moreover, environmental conditions in urban parks vary significantly, which may lead to some species adapting only to specific environments rather than a broader range of environmental conditions. For example, the Silver Pheasant (*Lophuranycthemera*) lives only in the Sanmenjiang Forest Park, while the Fairy Pitta (*Pittanympha*) lives only in the Dalongtan Park.

### Functional diversity

Interestingly, we observed the functional nestedness of all bird groups in Liuzhou. This implies that park size influences bird functional nestedness, with larger parks exhibiting greater functional trait variation. Smaller parks are less capable of maintaining and providing ecological functions than larger parks. Given that the area often serves as a good proxy for habitat diversity, the high degrees of trait diversity in large parks would simply be species with distinct traits occupying various habitats (Melo et al. 2014). Matthews et al. (2015b), using bird community data from 18 forest-habitat-island studies, found that most datasets (94%) were functionally nested by island area. Chen et al. (2022) found that the islands, characterised by large areas or high habitat diversity, had greater taxonomic and functional diversities of amphibians in the largest archipelago of China. Moreover, previous studies have demonstrated that the functional diversity (such as functional richness) of birds is positively correlated with the area of the park (Schütz and Schulze 2015). Therefore, large parks may have greater habitat diversity and richer niches, hence, more functional trait diversity. In general, different bird species have varying requirements for park areas (Tan et al. 2020). For example, birds requiring larger habitat areas may not be able to survive in smaller urban parks. In this sense, small urban parks may act as habitat filtering, where only species with similar traits can survive and reproduce in a given abiotic environment (Miller et al. 2016, Cadotte and Tucker 2017). To some extent, these traits are evolutionarily conserved, and habitat filtering can lead to local species assemblages that are more closely related than would be expected by chance (Miller et al. 2016). Moreover, in our survey, we found that smaller urban parks are more vulnerable to human disturbances, such as noise and pedestrian traffic. These human disturbances may potentially filter out bird functional traits that are not adapted to disturbed environments (Mouillot et al. 2013), which could also lead to the functional nestedness of bird species in urban parks.

We observed treeNODF values were primarily represented by S.Fraction rather than the topoNODF component. Previous studies have demonstrated that the observed functional nestedness of bird datasets was more a result of the species composition of islands than the functional differences between species (Matthews et al. 2015b). Similarly, the same pattern has been observed, for example, in bats (Melo et al. 2014), amphibians (Chen et al. 2022) and mammals (Millien et al. 2024). Even so, topoNODF represented a sizeable proportion of traitNODF for a number of bird datasets (Matthews et al. 2015b), indicating that the inclusion of functional traits into nestedness analyses can be revealing in certain instances.

## Conclusions

Comprehending the nestedness pattern and the underlying causal mechanisms holds paramount ecological significance for safeguarding biodiversity and effectively guiding management efforts (Melo et al. 2014, Matthews et al. 2015b). In this study, we found that the choice of null model had a significant impact on the results. We did not find a significant nestedness pattern of taxonomic diversity. However, as with the non-synchronicity of functional and taxonomic diversity, we observed significant functional nestedness, implying that the loss of traits from parks of decreasing area is proceeding in an ordered manner. That is, species with unique traits are being lost systematically (Matthews et al. 2015b). This finding suggests that species with specific functional traits are more vulnerable to habitat loss and fragmentation. To enhance biodiversity conservation in Liuzhou, we recommend prioritizing the protection of larger urban parks or creating interconnected green spaces to support species with larger habitat requirements. Additionally, conservation efforts should focus on restoring species with unique functional traits that are systematically lost from smaller parks. All in all, by considering both taxonomic and functional nestedness, we can achieve a more comprehensive understanding of nestedness processes and improve our capacity to conserve diverse aspects of biodiversity.

## Supplementary Material

A898DD46-2290-54F2-BC36-72611BFB178F 10.3897/BDJ.13.e154385.suppl1 Supplementary material 1 Bird species list in Liuzhou of Guangxi, China Data type Abundance File: oo_1326603.xlsx https://binary.pensoft.net/file/1326603 Binqiang Li
